# A multimodal deep learning model for predicting severe hemorrhage in placenta previa

**DOI:** 10.1038/s41598-023-44634-1

**Published:** 2023-10-13

**Authors:** Munetoshi Akazawa, Kazunori Hashimoto

**Affiliations:** Department of Obstetrics and Gynecology, Tokyo Women’s Medical University Adachi Medical Center, Adachi-ku, Kohoku 2-1-10, Tokyo, Japan

**Keywords:** Experimental models of disease, Reproductive disorders, Risk factors

## Abstract

Placenta previa causes life-threatening bleeding and accurate prediction of severe hemorrhage leads to risk stratification and optimum allocation of interventions. We aimed to use a multimodal deep learning model to predict severe hemorrhage. Using MRI T2-weighted image of the placenta and tabular data consisting of patient demographics and preoperative blood examination data, a multimodal deep learning model was constructed to predict cases of intraoperative blood loss > 2000 ml. We evaluated the prediction performance of the model by comparing it with that of two machine learning methods using only tabular data and MRI images, as well as with that of two human expert obstetricians. Among the enrolled 48 patients, 26 (54.2%) lost > 2000 ml of blood and 22 (45.8%) lost < 2000 ml of blood. Multimodal deep learning model showed the best accuracy of 0.68 and AUC of 0.74, whereas the machine learning model using tabular data and MRI images had a class accuracy of 0.61 and 0.53, respectively. The human experts had median accuracies of 0.61. Multimodal deep learning models could integrate the two types of information and predict severe hemorrhage cases. The model might assist human expert in the prediction of intraoperative hemorrhage in the case of placenta previa.

## Introduction

Placenta previa occurs in 4 per 1000 births, and the management of cesarean section for placenta previa is a major obstetric problem^1,2^. Since placenta previa sometimes causes massive hemorrhage during cesarean section, preoperative preparation including blood transfusion or interventional radiology, such as uterine artery embolization and intra-aortic balloon occlusion, are important. However, in some cases, blood loss is within normal limits without blood transfusion, and accurate prediction of severe hemorrhage in cases of placenta previa is desired in clinical situations. Accurate prediction leads to the classification of bleeding risk, which in turn leads to the allocation of medical and human resources.

Currently, machine learning and deep learning models have been extensively studied in various fields as new techniques for predictive and diagnostic tools. Among the various types of machine learning, deep learning has been implemented in a wide range of sciences and has become increasingly popular in recent years. Most models process a single type of information from images, sounds, text, audio, or tabular data. However, these models cannot cover all aspects of human learning. Multimodal learning helps to better understand and analyze when various senses are engaged in the processing of information^3^. In clinical situations, human clinicians can gather multiple pieces of information (e.g., patients’ biographs, blood examination, and imaging examination), following which the prognosis can be predicted (e.g., response to the treatment and possibilities of intraoperative complications). In the medical field, researchers have studied multimodal deep learning models, which can process and link information using various types of information, such as images, text, and tabular data^4–6^. Multimodal learning model can integrate multiple types of information to produce a better prediction.

In this study, we investigated a multimodal deep learning model for predicting massive hemorrhage during cesarean section for placenta previa, using patient demographics and preoperative MRI images.

## Materials and methods

### Patient enrollment

A total of 48 cases of placenta previa were enrolled, all of which were delivered at our tertiary perinatal medical center between 2008 and 2022. All patients were diagnosed with placenta previa by transvaginal ultrasound and underwent preoperative MRI during admission. In this study, severe hemorrhage was defined as an intraoperative hemorrhage ≥ 2000 ml or more. Bleeding was defined as the sum of the amount of blood lost through the gauze and suction tube.

**Figure 1:**
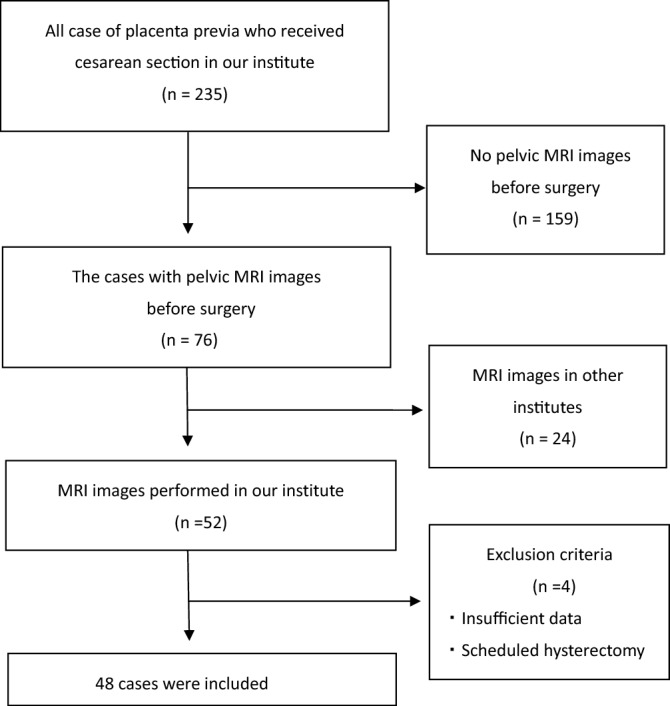
The flow chart of patients’ selection.

The inclusion criterion for women was cesarean section under the diagnosis of placenta previa. Emergency cesarean sections were also performed. We included women who underwent both pelvic MRI examinations and cesarean sections at our institute. The exclusion criteria were cesarean section in which scheduled hysterectomies were performed without extraction of the placenta. Twin cases, deliveries before 22 weeks, and cases lacking patient information were also excluded (Fig. 1). The study was approved by the institutional review board (IRB) of Tokyo Women’s Medical University. Due to the retrospective nature of the study, the IRB of Tokyo Women’s Medical University waived the need of obtaining informed consent. This study was designed and conducted in accordance with the relevant guidelines and regulations of the ethical principles for medical research involving human subjects, as stated in the WMA Declaration of Helsinki.

### Clinical variables, MRI images and target of prediction

As input data for the model, we used two types of data: tabular data, including patient demographics and blood examinations, and imaging data, including preoperative MRI images.

Tabular data included nine variables: (1) age, (2) number of pregnancies (gravity), (3) number of deliveries (parity), (4) number of previous cesarean sections, (5) number of gestational weeks at delivery, (6) birth weight, (7) hemoglobin, (8) white blood cell count, and (9) platelet count in blood examination before cesarean section. For each variable, we performed statistical analysis and divided the patients into two groups. In the statistical comparison of the two groups; Student’s t-test was used to analyze significant differences in quantitative parameters. Statistical significance was set at *p* < 0.05. We also calculated the Pearson correlation coefficient of each variable against the bleeding outcome to measure the impact of the variables.

**Figure 2:**
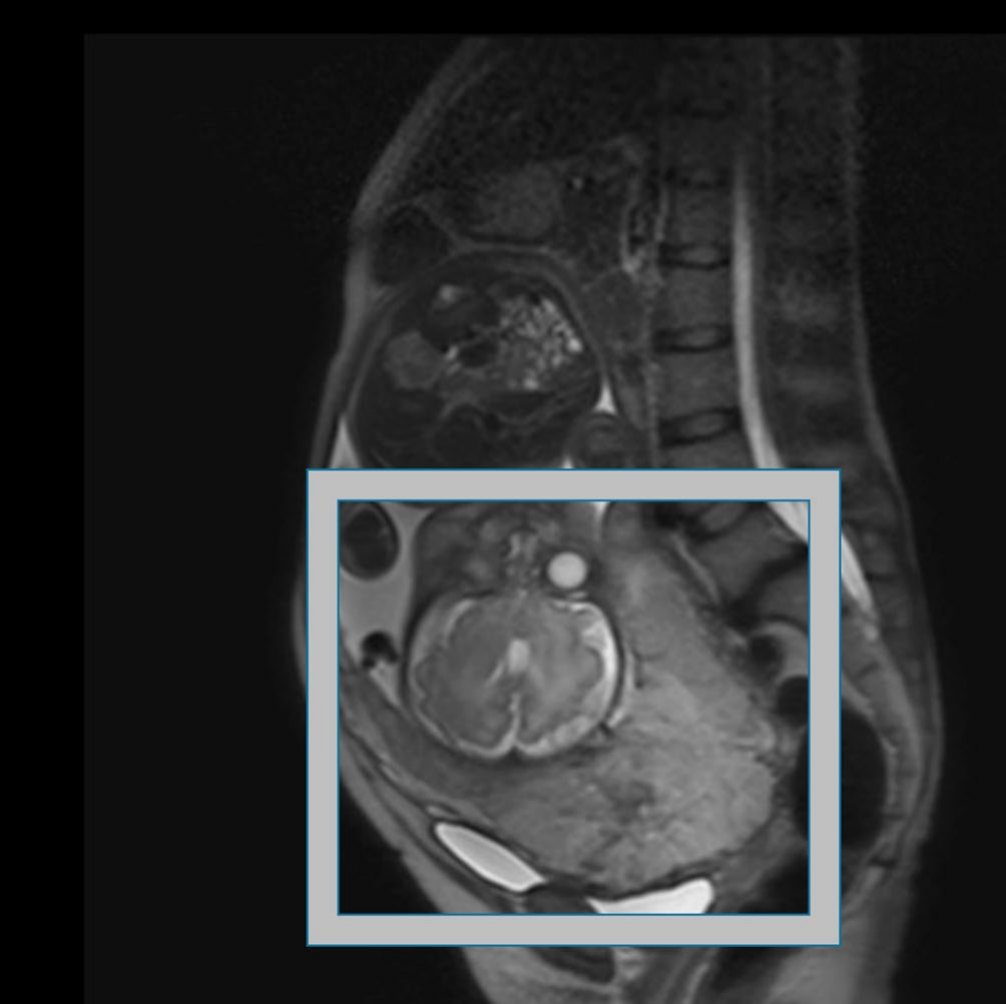
An example of MRI image of placenta previa. In this case, the placenta covered the anterior wall and os of the uterus, which was considered to cause massive bleeding during cesarean section. However, in this case, the blood loss was under 2000 ml. In the model construction, the field shown by the white bounding box was used for the learning.

Pelvic MRI was performed using a 3.0-T scanner (Achieva; Philips Medical Systems, Netherlands) at our institution. As described above, MRI images from other institutes were excluded considering their impact on digital images under different conditions. Among the series of MRI images, we used sagittal MRI T2-weighted images (T2WI) because obstetricians frequently use them in clinical situations. While we used a single image for each patient, the selection criteria for a single image were as follows: 1) the internal os of the uterus was included; and 2) if the internal os of the uterus could not be detected, the image with the full uterine cervix was included. The images were selected by an obstetric expert (M.A.), followed by a quality check by another obstetric expert (K.H.). In this image selection process, the human experts were blinded to which images belonged to cases of intraoperative blood loss > 2000 ml. After a single image was selected, the region of interest (ROI) was manually extracted using a bounding box. For the ROI in this study, we extracted a rectangular region, including of the entire placenta, internal os of the uterus, and uterine myometrium where the placenta was attached (Fig. 2). We believe that both the placenta and the uterine myometrium might have features related to bleeding. These MRI T2-weighted image (T2WI) sagittal images were divided into training and validation data at a ratio of 8:2.

As the target of prediction, severe hemorrhage was defined as blood loss of ≥ 2000 mL during loss. Blood loss through the suction tube and the count of gauze used were calculated as blood loss during cesarean section.

### Construction of models

**Figure 3:**
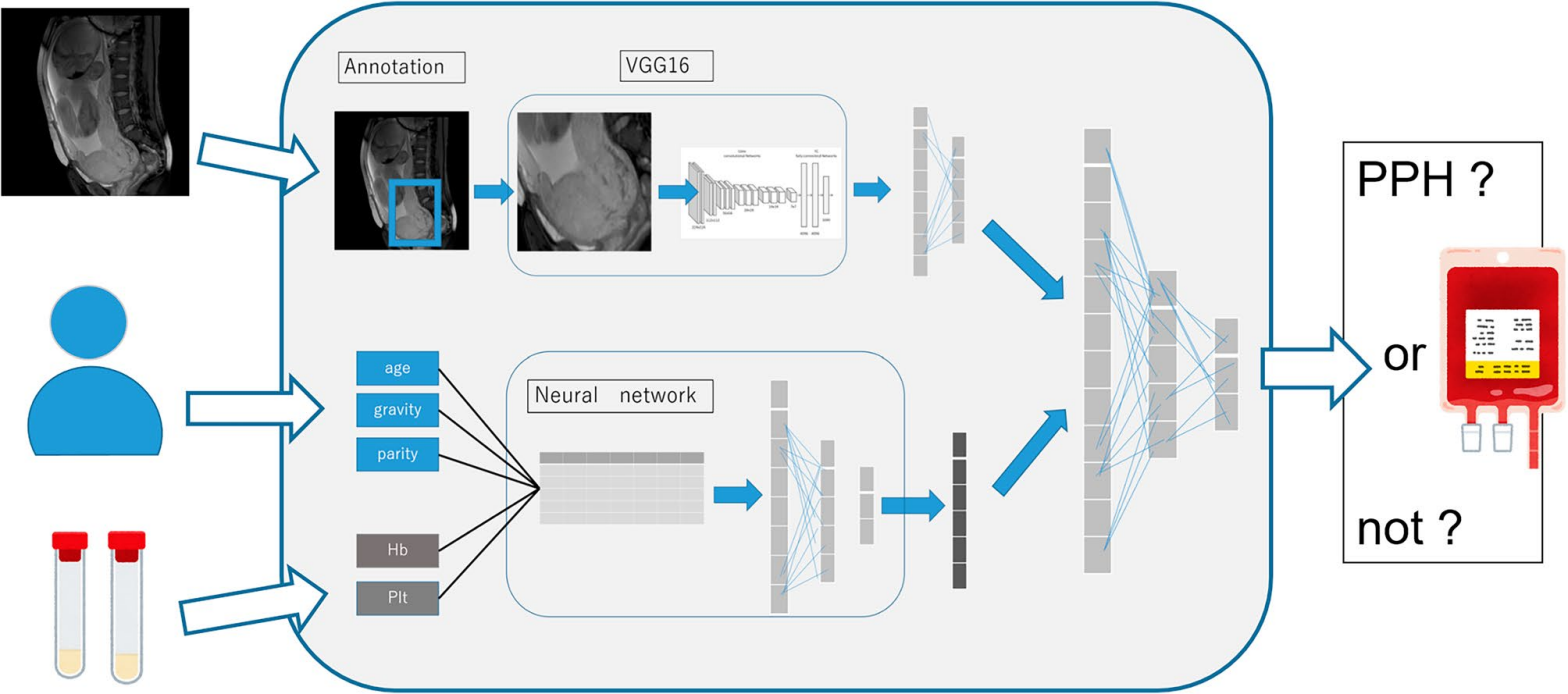
The pipeline of the multimodal deep learning model (Model 1).

We constructed a multimodal deep learning model (Model 1, shown in Fig. 3), consisting of two neural networks: the convolutional neural network for the MRI image and two layers of neural network for the tabular data. Obtaining the two inputs from these neural networks, an additional neural network outputs the prediction results. For MRI images, we constructed a VGG16-based model as a pre-trained convolutional neural network and extracted the features of the images, outputting 256 vectors. For the tabular data, the input was 12 variables and the output was six vectors through the two hidden layers. For the feature fusion, we combined two one-dimensional vectors extracted from the convolutional neural network (CNN) model and tabular data processed through a neural network and concatenated them. After concatenating these two vectors, we added three layers of neural networks to produce a prediction output. For the hyper-parameters, we used a rectified linear unit as the activation function, cross-entropy loss as the loss function, and stochastic gradient descent as the optimization method. The input image data were 224 × 224 and the batch size was set to 32. The CNN models were trained using pre-trained VGG16 weights (ImageNet dataset) for 100 epochs using an early stopping method.

**Figure 4:**
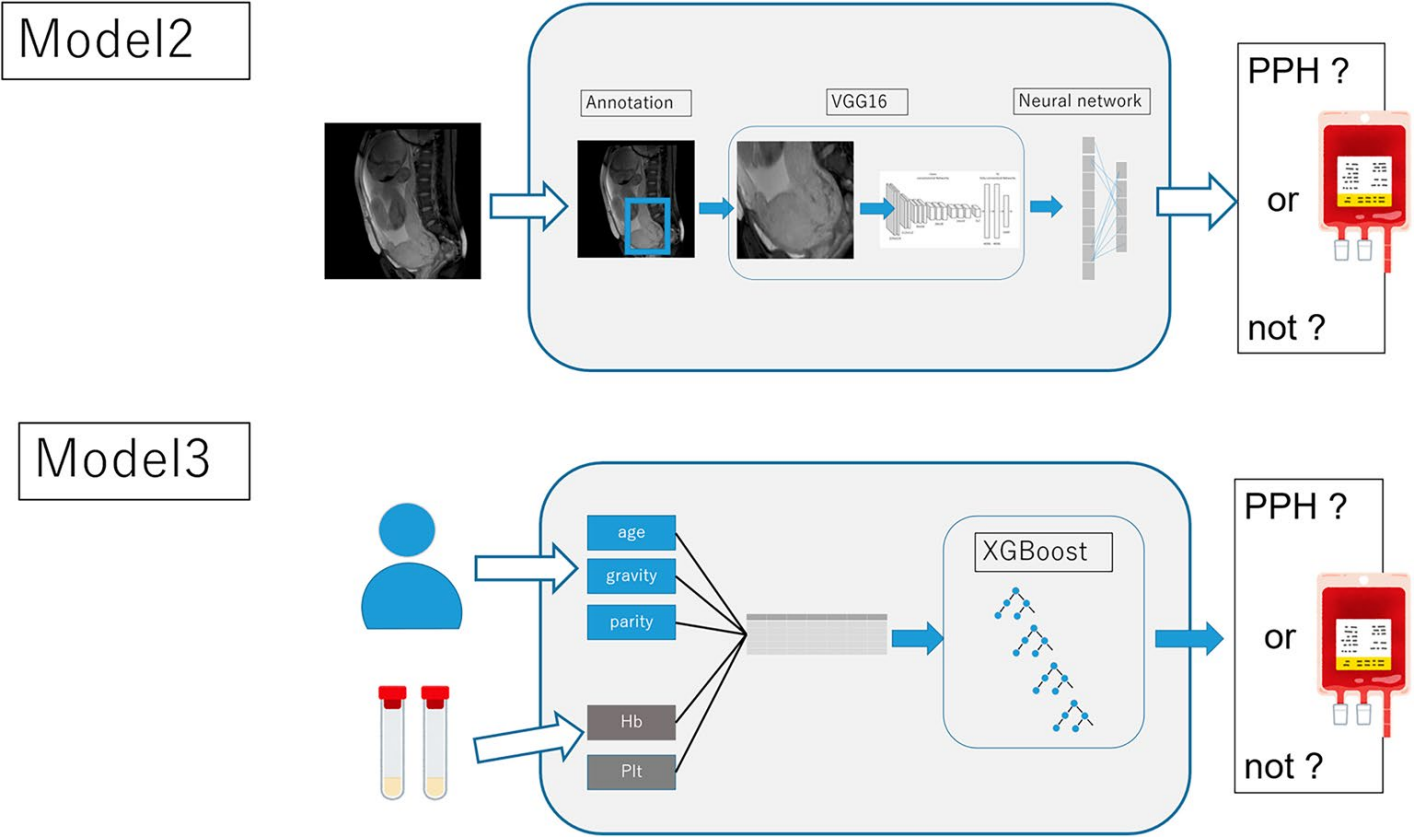
The pipeline of the two models using only tabular data and imaging data (Model 2 and Model 3).

Along with the multi-modal deep learning model, we constructed two models using only tabular data and imaging data (Models 2 and 3, shown in Fig. 4). For tabular data, we constructed a machine learning model based on the gradient boosting machine (XGboost). We also constructed VGG16-based models for the MRI images. All data were randomly divided into training and validation data at a ratio of 8:2, and cross-validation was used for 1:4 cross-validation. Regarding simple VGG16-based models, the hyper-parameters were described in a manner similar to that of the multimodal deep learning model. In the machine learning model using XGboost, the hyper-parameters were as follows: max_depth, 4; learning_rate, 0.1; and n_estimators, 120. The objective was binary logistic regression and the corresponding learning objective was binary logistic regression.

### Evaluation

For the evaluation of variables in tabular data, we calculated the Pearson correlation coefficient of each variable against the bleeding outcome to measure the impact of the variables. Pearson’s correlation coefficient for each variable was calculated as follows:

$$ {\text{r}} = \frac{{{\text{n}}\left( {\sum {{\text{xy}}} } \right) - \left( {\sum {\text{x}} } \right)\left( {\sum {\text{y}} } \right)}}{{\left[ {{\text{n}}\sum {{\text{x}}^{2} } - \left( {\sum {\text{x}} } \right)^{2} } \right]\left[ {{\text{n}}\sum {{\text{y}}^{2} } - \left( {\sum {\text{y}} } \right)^{2} } \right]}} $$

r = Pearson coefficient, n = number of pairs of the stock, ∑xy = sum of products of the paired stocks, ∑x = sum of the x scores, ∑y = sum of the y scores, ∑x^2^ = sum of the squared x scores, ∑y^2^ = sum of the squared y scores

Prediction accuracy was evaluated in terms of class accuracy, and the area under the curve (AUC). Class accuracy was calculated as follows:

$$ {\text{A}}\;{\text{class}}\;{\text{accuracy }} = \, \left( {{\text{TP}} + {\text{TN}}} \right) \, / \, \left( {{\text{TP}} + {\text{TN}} + {\text{FP}} + {\text{FN}}} \right) $$

TP, true positive; TN, true negative; FP, false positive; FN, false negative.

The point estimates for each metric were calculated by testing each model on the validation dataset. We generated 95% confidence intervals and p-values using the empirical bootstrap method with 1000 iterations. Student’s *t*-test was used to analyze significant differences in the quantitative parameters. Statistical significance was set at *p* < 0.05. Statistical analyses were performed using Python (version 3.7). Machine learning and deep learning were implemented using Keras (version 1.2.2) and SKlearn (version 1.0) in Python (version 3.7) as a programming language.

Finally, we tested two obstetrics experts at our institute, allowing them to observe the MRI images and tabular data together. The human experts did not need training; thus, all data were used for the test and evaluated by class accuracy only.

## Results

### Patient background

The patients’ background and statistical analysis of each variable are shown in Table 1. Among the 48 patients, 26 (54.2%) had blood loss > 2000 ml (positive group) and 22 (45.8%) had blood loss < 2000 ml (negative group). The overall mean blood loss was 2510 ml (maximum, 9664 ml; minimum, 602 ml) and that in the positive and negative groups was 3536 ml and 1292 ml, respectively. The mean age was 35.9 years, and the median times of pregnancy, delivery, and previous cesarean section were 1.6, 1.1, and 0.27 times respectively. Regarding patients’ backgrounds, there were no statistical differences between the positive and negative groups. However, the duration of pregnancy was higher in the positive group (*p*-value = 0.055). The mean pregnancy week for labor was 35.8 weeks of gestation, and the median birth weight of the baby was 2609 g. In the preoperative blood examination results, there was no statistical difference between the positive and negative groups. Among the variables, while statistical significance was set at *p* < 0.05, no differences were observed in this study.

**Table 1:** The patient’s background.

	Total	PPH	non-PPH	*p*-value	Correlation coefficient
Number of cases	48	26	22		
Age (yr)	35.9 (24–43)	35.7	36.0	0.82	− 0.033
Gravity	1.6 (1–6)	1.9	1.3	0.055	0.278
Parity	1.1 (0–4)	0.92	1.2	0.22	− 0.179
The frequency of previous CS	0.27 (0–2)	0.27	0.27	0.98	− 0.003
WBC	8087 (4900–12,100)	8311	7822	0.39	0.125
Hemoglobin (g/dl)	10.7 (9.1–11.9)	10.7	10.6	0.51	0.097
Platelet	23.4 (8.8–41.2)	24.1	22.6	0.48	0.103
Gestation of delivery (week)	35.8 week (30–38)	35.4	36.2	0.051	− 0.283
Weight of neonate (g)	2609 g(1150–3260)	2531	2700	0.17	− 0.201
The amount of bleeding (ml)	2510 ml (602–9664)	3536	1297	< 0.001	

*CS* cesarean section, *PPH* postpartum Hemorrhage, *WBC* white blood cell.

In the analysis of correlation coefficients, while coefficients were set above 0.2, the important variables were gravity, weight of the neonate, and gestation week. Similarly, these variables showed higher t-statistics in the results of the statistical significance analysis. The frequency of previous cesarean section, which is considered an important factor leading to placenta accreta spectrum (PAS), showed no significant difference and a low correlation coefficient in this study.

### Prediction performance

**Figure 5:**
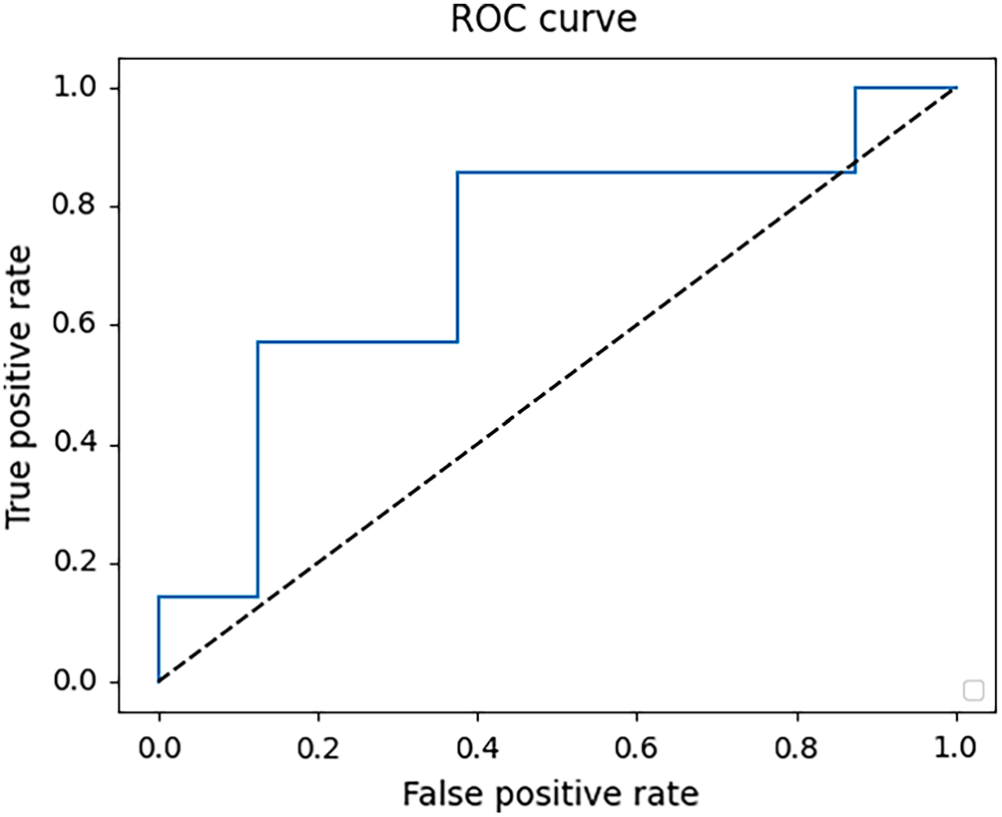
ROC curve of the multimodal deep learning model.

Table 2 shows the prediction results for each model and the human experts. Multimodal deep learning model showed the best accuracy of 0.680 [95% confidence interval (95%CI) 0.640–0.703], whereas the machine learning model using tabular data and MRI images had a class accuracy of 0.610 (95% CI 0.533–0.716) and 0.537 (95% CI 0.476–0.601), respectively. Regarding AUC, multimodal deep learning model also showed the best AUC of 0.735 (95% CI 0.668–0.800), whereas the machine learning model using tabular data had a AUC of 0.576 (95% CI 0.447–0.700). We showed ROC curve of multimodal deep learning model (Fig. 5).

**Table 2:** The comparison of prediction performance.

Model	Accuracy	AUC
Model 1: Multimodal deep learning (Tabular + Image)	0.680 (0.640–0.703)	0.735 (0.668–0.800)
Model 2: Machine learning (Tabular)	0.610 (0.533–0.716)	0.576 (0.447–0.700)
Model 3: Deep learning (Image)	0.537 (0.476–0.601)	
Human expert	0.614	

*AUC* area under the curve, *95% CI* 95% confidence interval.

Although the statistical difference was not shown in the tabular data, the model using the tabular data had better prediction performance than the model using imaging data. Human experts could not be evaluated using the AUC. Using all the data for the test, the two human experts had mean accuracies of 0.614.

## Discussion

This study showed that multimodal deep learning models can predict severe hemorrhages better than human experts and machine learning models using single data types. Although the size of the dataset was too small and future studies are required to verify our findings, this study demonstrated that multimodal deep learning integrating clinical information could lead to better predictive performance (accuracy, 0.68). Trends in improvements using multimodal models were reported previously. Reporting a multimodal model for brain tumors, Artzi et al. commented that better performance by the fusion of medical images and patient records was consistent with the results of other studies of melanoma, breast cancers, and brain tumors^6^. In clinical situation, the human experts performed the same predictions by analyzing multiple factors. Obstetricians empirically predict intraoperative bleeding by integrating several factors such as the number of previous cesarean sections, patient age, anemia on a blood examination, and placental position on MRI or ultrasound examinations. Although the predictive importance of each variable was low, the integration of multiple types of information was essential. For the prediction of severe hemorrhage in obstetrics, our study was the first to use multimodal deep learning models, which showed better prediction performance than human experts and models using a single type of information.

This study presented several facts about the other models. Although clinical variables in demographics and preoperative blood examination data did not differ significantly in the statistical analysis, the machine learning model using these data showed a predictive accuracy of 0.61. In contrast, CNN models using images did not exhibit predictive performance (accuracy, 0.53). Although the CNN model could not sufficiently extract the features due to the small size of the dataset, the clinical variables might be useful for prediction to some extent. Moreover, our study demonstrated that human prediction is challenging (accuracy, 0.61). Clinically, human experts make the diagnosis of placenta previa to prepare for severe hemorrhage in cesarean sections. However, among the case of placenta previa, an additional risk classification of hemorrhage is currently lacking. Human predictive performance was low (accuracy, 0.61) and insufficient for decision-making in the management of placenta previa. Among the two human experts, the degree to which the prediction of the obstetrician matched was 0.77, while the degree to which the prediction of one human obstetrician and multimodal model matched was 0.60. This reason was considered to be because the human experts had the similar trends of prediction; the case with history of previous cesarean section or anterior position of placenta fully covering os of the uterus was thought to be the positive cases of hemorrhage. And the feature extraction among the human and deep leaning might be different. Since the augmentation of data could enhance the predictive accuracy of artificial intelligence (AI) systems, multimodal deep learning using large datasets might show predictive accuracy of 0.7–0.8 in future studies.

In obstetrics, there are few numerical markers that predict prognosis, such as clinical stage or tumor markers, for cancer management. No numerical markers are currently used clinically to predict or classify risk of obstetric hemorrhage^7,8^. If the possibility of severe hemorrhage can be output with numerical values using models, risk classification can be performed preoperatively, leading to the optimum allocation of interventions such as medical staff, blood transfusion, and interventional radiology. A predictive model and risk stratification for obstetric bleeding are needed to save mothers worldwide.

AI enables creation of novel predictive and diagnostic techniques. Along with advances in computer science and technology, research on AI in the medical field has spread rapidly in recent year. Multimodal deep learning models, which process several types of clinical data, such as pathology, imaging data, and patient demographics, have been studied for better performance^4–6^. Considering that physicians make decisions by integrating various data in the patient, such a multimodal model could improve the prediction performance. In the field of placentas, a few studies using deep learning have been published^9–11^. Romeo et al. reported a prediction model that predict placenta accrete spectrum (PAS) in patients with placenta previa^9^. Using preoperative MRI examinations of 64 patients with placenta previa, they extracted rounded regions of interest manually positioned on T2WI MRI image of the placenta. Their machine-learning model achieved an accuracy of 0.98. Liu et al. reported a prediction model that predicts intraoperative bleeding of > 500 ml in patients with placenta previa^10^. Using the preoperative MRI examinations of 210 patients, including 78 placenta previa cases, they constructed a VGG16-based model on T2WI MRI images of the placenta. Their deep learning models achieved an accuracy, sensitivity, and specificity of 0.75, 0.73, and 0.77, respectively.

The strengths of this study are as follows. This was the first study on multimodal deep learning for placental research. Although the size of the dataset was small, we demonstrated the possibilities of multimodal deep learning for this clinical theme. Both imaging and numerical data of patients have an impact on the prediction of massive bleeding, and such a model combining several types of data is desired. Additionally, we showed the difficulties of predicting massive bleeding in human beings. While the case of placenta previa placed in the anterior wall with a history of cesarean section is considered high-risk for severe bleeding, in other cases, there is a lack of methods to predict high-risk cases of placenta previa. In the statistical evaluation of the two groups, the statistical differences in patient demographics and blood examinations were small. As such, when deep learning can extract hidden features in large datasets and imaging data, there is the possibility of more accurate prediction using the hidden features through deep learning.

This study’s limitations and future challenges are as follows. First, the small size of the dataset presents a major challenge. Without a large dataset, deep learning cannot be adequately trained. Moreover, because the validation data were also small in such a situation, the validation itself could be unstable, owing to the random-based evaluation of machine learning. Second, external validation data were not used in this study. The machine learning model could have an overfitting problem, and internal validation could cause overfitting. A larger dataset and another dataset for validation are desired for deep learning research. Third, the manual segmentation of MRI images is controversial. While we used part of the placenta around the os and anterior wall in this study, it is still unknown which site in the placenta images could have important features for the prediction of severe hemorrhage. When deep learning studies on placental imaging progress, a more suitable placental image site can be found and learning can be promoted. Fourth, we did not use ultrasound images as an image of the placenta. In clinical practice, ultrasound examination is the gold standard for the diagnosis of placenta previa. However, ultrasound data are not unique and have noise because ultrasound is performed by humans compared with MRI. Therefore, we used MRI images in this study. In contrast, ultrasound data are easier to gather than MRI data. Thus, for future AI studies, imaging sources should be analyzed in terms of uniformity and accessibility of images. Fifth, regarding the target of prediction, during analysis using AI, the unequal distribution of positive and negative groups disturbed the training, as AI could not learn on the small-sized data of positive groups. Thus, the prediction of PAS in placenta previa will be difficult unless data regarding a reasonable number of cases of PAS can be gathered.

Deep learning can be used to extract features for predicting clinical outcomes. In this study, we demonstrated the possibility of a multimodal deep learning model for the prediction of severe hemorrhage. In future studies, with a larger dataset and more efficient segmentation of MRI images, the prediction performance could be improved.

## Data Availability

The datasets analyzed during the current study are available from the corresponding author on reasonable request.
